# Influence of chronic azithromycin treatment on the composition of the oropharyngeal microbial community in patients with severe asthma

**DOI:** 10.1186/s12866-017-1022-6

**Published:** 2017-05-10

**Authors:** Guido Lopes dos Santos Santiago, Guy Brusselle, Kenny Dauwe, Pieter Deschaght, Chris Verhofstede, Dries Vaneechoutte, Ellen Deschepper, Paul Jordens, Guy Joos, Mario Vaneechoutte

**Affiliations:** 1 Laboratory Bacteriology Research, Department Clinical Chemistry, Microbiology & Immunology, Faculty of Medicine & Health Sciences, University of Ghent, Ghent, Belgium; 2 Department of Respiratory Medicine, Ghent University Hospital, Ghent, Belgium; 3 AIDS Reference Laboratory, Ghent University, Ghent, Belgium; 4 Department of Plant Systems Biology (VIB), Department of Plant Biotechnology and Bioinformatics, Ghent University, Ghent, Belgium; 5 Biostatistics Unit, Department of Public Health, Ghent University Hospital, Ghent, Belgium; 6 Department of Respiratory Medicine, OLV Ziekenhuis Aalst, Aalst, Belgium

**Keywords:** Asthma, Oropharyngeal microbiome, Azithromycin, Antibiotic treatment, Oral microbiome

## Abstract

**Background:**

This study of the oropharyngeal microbiome complements the previously published AZIthromycin in Severe ASThma (AZISAST) clinical trial, where the use of azithromycin was assessed in subjects with exacerbation-prone severe asthma. Here, we determined the composition of the oropharyngeal microbial community by means of deep sequencing of the amplified 16S rRNA gene in oropharyngeal swabs from patients with exacerbation-prone severe asthma, at baseline and during and after 6 months treatment with azithromycin or placebo.

**Results:**

A total of 1429 OTUs were observed, of which only 59 were represented by more than 0.02% of the reads. Firmicutes, Bacteroidetes, Fusobacteria, Proteobacteria and Actinobacteria were the most abundant phyla and *Streptococcus* and *Prevotella* were the most abundant genera in all the samples. Thirteen species only accounted for two thirds of the reads and two species only, *i.e. Prevotella melaninogenica* and *Streptococcus mitis/pneumoniae,* accounted for one fourth of the reads.

We found that the overall composition of the oropharyngeal microbiome in patients with severe asthma is comparable to that of the healthy population, confirming the results of previous studies. Long term treatment (6 months) with azithromycin increased the species *Streptococcus salivarius* approximately 5-fold and decreased the species *Leptotrichia wadei* approximately 5-fold. This was confirmed by Boruta feature selection, which also indicated a significant decrease of *L. buccalis*/*L. hofstadtii* and of *Fusobacterium nucleatum*. Four of the 8 treated patients regained their initial microbial composition within one month after cessation of treatment.

**Conclusions:**

Despite large diversity of the oropharyngeal microbiome, only a few species predominate. We confirm the absence of significant differences between the oropharyngeal microbiomes of people with and without severe asthma. Possibly, long term azithromycin treatment may have long term effects on the composition of the oropharygeal microbiome in half of the patients.

**Electronic supplementary material:**

The online version of this article (doi:10.1186/s12866-017-1022-6) contains supplementary material, which is available to authorized users.

## Background

Asthma is a chronic disease of the airways, characterized by chronic airway inflammation and variable symptoms of wheezing, dyspnea and cough [1]. The genetic and environmental factors that determine asthma are not well understood, but several studies suggest that microbes from oral sites not only contribute to colonization of the airways in disease (as in cystic fibrosis) [2–4], but also that microbial colonization of the airways might have a role in the chronic inflammatory process [1, 5]. For instance, both *Chlamydophila pneumoniae* and *Mycoplasma pneumoniae* have been detected in respiratory secretions from patients with acute asthma exacerbations [6–8].

Recently, the macrolides erythromycin and azithromycin have been assessed as an addition to standard therapy to prevent exacerbations in patients with asthma or chronic obstructive pulmonary disease (COPD) [9–13]. Macrolides exert their antimicrobial effects by binding to the 50S ribosomal RNA subunit and have a broad spectrum of activity against many micro-organisms [14]. In addition, macrolides have multiple immunomodulatory and anti-inflammatory effects [12, 15]. However, use of antibiotics has been associated with the development of antimicrobial resistance of bacteria in individual patients and at population level. Although the induction of antimicrobial resistance has been investigated in healthy individuals after short-term administration of macrolides, the effects of chronic treatment with macrolides on the composition of the pharyngeal microbiome in patients with (severe) asthma remain to be elucidated.

In this study, we determined the composition of the oropharyngeal microbial community by means of deep sequencing of the amplified 16S rRNA gene in oropharyngeal swabs from patients with exacerbation-prone severe asthma at baseline and during treatment with azithromycin or placebo for 6 months. Samples had been collected during the AZIthromycin in Severe ASThma (AZISAST) randomized controlled clinical trial (RCT) [12].

## Methods

### Study background

This study of the oropharyngeal microbiome complements a previously reported clinical study, *i.e.* the AZIthromycin in Severe ASThma (AZISAST) trial [12], which was a randomized, double-blind, placebo-controlled, parallel-group multicenter study in subjects with exacerbation-prone severe asthma. Severe asthma was defined as in the European Respiratory Society and American Thoracic Society Severe Asthma guidelines [16], *i.e.*, adult patients with asthma, needing treatment with high dose inhaled corticosteroids (ICS) and long-acting beta2-agonists (LABA) (thus Global Initiative for Asthma - GINA - guideline step 4 or 5), who still had experienced two or more exacerbations in the previous year despite this maintenance treatment. Moreover, the diagnosis of severe asthma was made by a respiratory physician. During the selection of the patients (See Table 1 for patient characteristics), the exacerbation and smoking history were taken into account (See Additional file 1 for the AZISAST-trial baseline characteristics [12]). A medical history of at least two exacerbations (treated with oral corticosteroids) in the previous year was an inclusion criterion. Current smoking or a smoking history of more than 10 pack years were exclusion criteria of the AZISAST study. The majority of patients were never smokers; ex-smokers had smoked less than 10 pack years, and had stopped smoking for at least one year.

**Table 1:** Baseline characteristics

Characteristic	Placebo *N* = 5	Azithromycin *N* = 8
Sex (no. of subjects)		
Male	3 (60%)	3 (38%)
Female	2 (40%)	5 (62%)
Age (yr)		
Median, Range, IQR	57 (41;64), (48;62)	48 (19;60), (40;52)
Race (no. of subjects; %)		
Caucasian	5 (100%)	8 (100%)
Body-mass index		
Mean (SD)	31.5 (5.5)	26.0 (5.9)
Positive atopic status		
(no. of subjects; %)*	3 (60%)	6 (75%)
Severe asthma exacerbations requiring treatment with systemic corticosteroids		
*previous 12 months* (% of subjects)	5 (100%)	6 (75%)
*previous 12 months (no. [mean], SD)*	2.2 (0.4)	1.9 (1.5)
Lower respiratory tract infections requiring treatment with antibiotics		
*previous 12 months* (% of subjects)	4 (80%)	6 (75%)
*previous 12 months (no. [mean], SD)*	1.0 (0.7)	2.8 (1.9)
FEV_1_ prebronchodilator (% of predicted)		
Mean (SD)	87.6 (19.1)	87.9 (19.0)
FEV_1_/FVC ratio prebronchodilator		
Mean (SD)	60.4 (12.1)	66.9 (9.4)
FE_NO_ (ppb)		
Median, Range, IQR	12.8, (8;26.7), (10;20.9)	30, (13;54), (20.3;37.5)
Eosinophil count in blood (x10-9/liter)		
Mean (SD)	494.0 (420.9)	161.3 (100.2)
Daily dose of inhaled corticosteroid (BDP-equivalent) (μg)		
Daily dose	2000, (2000;3000),	2125, (1500;4000),
Median, Range, IQR	(2000;2500)	(2000;3625)
Use of oral prednisolone		
Regular use (% of subjects)	1 (20%)	2 (25%)
Daily maintenance dose (mg)		
Median, Range, IQR	2.5, (−;-), (−;-)	8.8, (7.5;10), (−;-)
Use of montelukast (leukotriene receptor antagonsist) (no. of subjects; %)	2 (40%)	2 (25%)

Legend: *Atopic status: based on skin prick tests; if skin prick test was not interpretable or not available, the atopic status is based on serum RAST for standard aero-allergens (house dust mite, animal dander [cat, dog], pollen [grass, tree] and Aspergillus fumigatus)

*Abbreviations*: *ACQ* asthma control questionnaire, *AQLQ* asthma quality of life questionnaire, *BDP* beclomethasone dipropionate, *IQR* interquartile range, *LRT* lower respiratory tract

The AZISAST study including this bacteriological substudy was approved by the central ethics committee of Ghent University Hospital, and was reviewed by the local ethics committees at each participating site (2008/445, IWT 070709). All patients provided written informed consent.

The oropharyngeal samples for the bacteriologic substudy of the AZISAST trial were obtained in two centers (Ghent University Hospital and OLV Hospital Aalst). For this study and manuscript, only samples of patients enrolled at Ghent University Hospital were examined, in order to minimize heterogeneity due to differences in sampling techniques. Indeed, all oropharyngeal samples at Ghent University Hospital were taken by one experienced study nurse using a standardized procedure.

Briefly, during the AZISAST study, the subjects received low-dose azithromycin (*n* = 55) or placebo (*n* = 54) as an add-on treatment to combination therapy of inhaled corticosteroids and long-acting β2 agonists for 6 months. After randomization, patients took one capsule of 250 mg azithromycin (prepared from capsules of Zithromax) or placebo once daily for 5 days and then one capsule of 250 mg azithromycin or placebo three times a week. The total treatment period was 26 weeks (until visit 6), with a study drug-free follow-up period of 4 weeks (washout period) [12].

### Study design

We included three groups of patients in the bacteriological substudy, *i.e.* 5 subjects of the placebo group and 8 azithromycin (AZ)-treated patients encompassing 5 AZ responders and 3 AZ non-responders. The AZ responders were patients in the active treatment arm (azithromycin) who had a more than 50% decrease in the rate of the primary outcome (*i.e.* asthma exacerbations) during the treatment phase of the AZISAST trial compared to the previous year (*i.e.* the year before enrollment in the trial).

The oropharyngeal microbiome was assessed at four different visits during the trial, *i.e.* at visit 2 (V2): at randomization (just before treatment), at visit 3 (V3): one month after the start of treatment, at visit 6 (V6): at the end of the 6 months treatment period and at visit 7 (V7): one month after the end of the treatment period (*i.e.* at the end of the washout period).

### Sample collection

Although sputum (spontaneous or induced), bronchial brushings or bronchoalveolar lavage samples could possibly demonstrate more pronounced effects of azithromycin treatment, these samples are more difficult to obtain, and invasive techniques (such as bronchoscopy for bronchial brushings or lavage) could induce asthma attacks in patients with severe asthma. Therefore, we have chosen to sample the oropharynx, since this sampling technique is well tolerated and safe, and could be performed at four different visits in all patients (in the two centers who participated in the AZISAST sub study). Moreover the oropharynx can be regarded as the best proxy for the lung microbiome [17–21].

Briefly, oropharyngeal samples were obtained by means of a swab firmly pressed over the tonsils and the posterior pharyngeal wall [22]. The jaws, teeth and gingiva were avoided when the swab was withdrawn.

### DNA extraction and sequencing

For DNA extraction, the oropharyngeal swab was immersed in physiological water and a volume of 200 μl of the suspension was transferred to a 2 ml tube, to which 200 μl of buffer (20 mM Tris–HCl, pH 8.0, 0.5% Sodium dodecyl sulfate) was added. Subsequently, 2 μl of mutanolysin (25 U/μl) was added and the mixture was incubated for 15 min at 37 °C. Next, 10 μl of a 25 mg/ml proteinase K solution was added and the mixture was incubated for 15 min at 55 °C. Finally, NucliSENS EasyMAG lysis buffer was added to a final volume of 2 ml, and incubated for 10 min at room temperature. DNA extraction was performed on the NucliSENS EasyMAG (BioMérieux, Marcy l’Etoile, France) platform, according to the manufacturer’s instructions.

The bacterial 16S rRNA gene was amplified, starting from 25–100 ng of DNA template in the presence of 0.2 μM of the 27 F forward primer (AGAGTTTGATCMTGGCTCAG) and an equimolar mixture of the reverse primers 338R-B-I (GCWGCC**T**CCCGTAGG**A**GT) and 338R-B-II (GCWGCC**A**CCCGTAGG**T**GT) (to compensate for mismatches), specific for the V1-V2 hypervariable region of the bacterial 16S rRNA gene [23]. The forward primer had an adaptor sequence and a unique barcode sequence, *i.e.* MID (Multiplex IDentifier), to help identify the samples after sequencing. PCR products were visualized on agarose gel and purified using a double purification protocol with AMpure XP DNA-binding magnetic beads (Agencourt, Beckman Coulter, Woerden, the Netherlands), and quantified in triplicate, using the Qubit 2.0 dsDNA HS Assay (Life Technologies, Ghent, Belgium). Samples were then diluted to a concentration of 1 × 10^9^ molecules/μL and equimolar concentrations of 10 samples were pooled to create a multiplexed amplicon library. This amplicon library was purified again using a double purification protocol with AMpure XP DNA-binding magnetic beads. The library was further diluted to 10^6^ molecules/μl and subjected to emulsion PCR (emPCR – 35 PCR cycles instead of 50 cycles) using the 454 GS Junior Titanium Series Lib-L emPCR Kit (Roche Diagnostics, Vilvoorde, Belgium) and the Live Amp Mix B for Paired End libraries, according to the manufacturer’s protocols. After the emPCR, the DNA-bound beads were enriched with a second DNA capture mechanism to separate the beads with bound emPCR products from the empty beads. Using a bead counter, the number of enriched beads was estimated to be 500,000. The enriched pool of beads was then used for massive parallel pyrosequencing in a Titanium PicoTiterPlate with Titanium reagents (Roche Diagnostics), on the GS Junior instrument (454 Life Sciences, Branford, Connecticut), according to the 454 GS Junior Titanium Series Amplicon Library Preparation Method Manual.

### Sequence analysis

The Roche Amplicon filter pipeline was used for quality-filtering of the amplicons and for generating the Standard flowgram format (sff)-files. For the analysis of the sequencing data, the Genboree Microbiota Toolset was used as described by Riehle et al. [24, 25]. This toolset has integrated open source tools for 16S rRNA gene analyses, such as the Ribosomal Database Project (RDP) classifier [26] and QIIME (Quantitative Insights Into Microbial Ecology) [27].

The Genboree Workbench was used to upload the sequencing sff-files for each sample together with the associated sample metadata. Individual sequencing files were passed through a quality filtering algorithm that used the following filter read settings: Trim at distal primer, remove sequences with ambiguous nucleotides, minimum read length of 300 nt, minimum average quality score = 20 and minimum sequence count per sample = 1000. Subsequently, Operational Taxonomic Units (OTU) generation and analyses of alpha diversity and beta diversity, phylogenetic analysis, and feature selection were performed [24].

Briefly, OTU generation was accomplished by a multi-step OTU picking algorithm that generates representative sequences, whereby similar sequences are binned together into the same OTU, and produces an OTU table as a result. The OTU tables were used for downstream analyses, such as alpha diversity and beta diversity calculation, classification by supervised machine learning, and feature selection [24]. Beta diversity clustering plots (via Principal Coordinates Analysis (PcoA)) were generated using the phylogenetic unweighted UniFrac metric to determine the differences between the different clinical metadata groups [24, 28]. To confirm actual clustering of microbiomes on the basis of treatment (azithromycin vs. placebo) or on the basis of different visits, supervised learning with randomForest classification [24, 29] was used, with the assumption that if randomForest generated an estimated error rate of < 5%, it could be claimed that these clinical metadata groups contain significantly different microbial communities. In case of estimated error rates greater than 10%, Boruta feature selection still can be used to obtain statistically significant features [24, 30, 31]. In the supervised machine learning analyses, *i.e.* randomForest and Boruta feature selection, cut off values based on the sum of the rows in the OTU table were deployed for a variety of reasons, but mainly to remove noise and to decrease the time and resource requirements to run randomForest and Boruta. Removing low abundant OTUs has been found to increase the success of classifying (randomForests) and feature selection (Boruta) by decreasing the noise of the microbial community data representation. A range of cut off values, *i.e.* 5, 25, 100 and 500, was used to vary the degree of noise removal. For instance, a cut off value of 5 indicates that the OTUs (*i.e.* rows) from the OTU table that do not sum up to at least 5 reads are not taken into account [24, 31]. This allows to increase the weight of the highly abundant OTUs. The Human Oral Microbiota Database (HOMD [32], which provides a curated, full length, 16S rRNA gene reference data set [33, 34], was used to get an idea of which species the OTUs possibly represented. Species level identification was obtained through the HOMD Blast search tool, with a minimum alignment requirement of 97% with the query sequence [32]. Each species-level identified OTU was automatically assigned a human oral taxon (HOT) number.

## Results

### Background

Recently, Brusselle et al. [12] reported that patients with severe asthma and non-eosinophilic inflammation (normal Fractional exhaled nitric oxide-FeNO and blood eosinophilia < 200/μL) had a significant reduction of exacerbation rate upon treatment with low dose azithromycin (AZ) for 6 months, compared with the placebo arm (AZISAST trial). In contrast, patients with severe asthma and eosinophilic inflammation (blood eosinophilia > 200/μL) did not benefit from AZ treatment compared with placebo [12]. In the AZISAST-trial, bacterial cultures were performed on the oropharyngeal samples used in the present study, demonstrating an increased resistance of oropharyngeal streptococci to macrolides in the azithromycin-treated patients [12].

In this microbiological substudy of the AZISAST trial, we carried out a cross-sectional and longitudinal evaluation of the oropharyngeal microbiomes of the AZ-treated and placebo-treated groups by means of a cultivation-independent manner using next-generation sequencing of amplified 16S rRNA genes. We studied three groups of asthmatic patients, *i.e.* azithromycin (AZ) responders (*n* = 5; patients 106, 115, 126, 129 and 142), AZ non-responders (*n* = 3; patients 110, 122 and 151) and patients receiving placebo (*n* = 5; patients 101, 104, 111, 112 and 121).

We established the composition of the oropharyngeal microbiome and studied to what extent long term azithromycin treatment influenced the microbial composition of the oropharynx.

### Overall analysis

A total of 473.995 reads were obtained for 52 oropharyngeal samples (4 sampled visits from each of 13 subjects), of which 466.401 reads passed quality filtering. On average, there were 8.969 quality filtered reads per sample (range: 2.653–25.809 reads). All sequences are available at National Center for Biotechnology Information (NCBI) Bioproject with identifier PRJNA356972 [35].

Overall, a total of 1429 OTUs were recognized. Of these, only 59 (4%) were represented by 0.2% or more of the reads, accounting for 89.75% of all the reads (Additional file 2). Most of these 59 OTUs correspond to HOMD Human Oral Taxons (HOTs), *i.e.*, species with familiar names in oral microbiology [34], but some of these species, such as *Lachnoanaerobaculum orale* (0.37% of overall number of reads), *Lautropia mirabilis* (0.46%), *Megasphaera micronuciformis* (0.72%), *Oribacterium sinus* (0.57%), *Solobacterium moorei* (0.36%) and genus TM7 [G-1] sp. (0.38%) from the phylum Saccharibacteria, are not as well-known. On the other hand, species such as *Haemophilus influenzae* and *Moraxella catarrhalis*, well-known inhabitants and potential pathogens of the upper airways, were virtually absent. *M. catarrhalis* was found in only one sample (Patient 106 Visit 2), representing only 0.06% of the reads of that sample. *H. influenzae* was present in 17 samples (7 V2 samples, 5 V3 samples and 5 V7 samples), but represented on average only 0.02% of the reads per sample (range 0.01–0.07%). The only exception was *Haemophilus parainfluenzae,* a potential pathogen and common inhabitant of the oropharynx, accounting for 2.11% of the reads. The presence or absence of *Streptococcus pneumoniae* is difficult to establish, because of the high similarity with the closely related commensal *S. mitis* (accounting for 10.68% of the reads) in the V1-V2 region that was sequenced for this study. No reads for *Chlamydophila pneumoniae* or *Mycoplasma pneumoniae* were present among the asthma patients, although *M. faucium*, *M. lipophilum* and *M. salivarium* were recovered in low numbers.

Moreover, only 13 species (HOTs) accounted for 65.03% of the reads, *i.e.*: *Prevotella melaninogenica* (HOT-469; 15.17%), *Streptococcus mitis/pneumoniae* (HOT-677; 10.68%), *Streptococcus parasanguinis* (HOT-411; 6.84%), *Veillonella atypica* (HOT-524; 5.41%), *Streptococcus salivarius* (HOT-755; 4.13%), *Leptotrichia wadei* (HOT-222; 3.86%), *Granulicatella adiacens* (HOT-534; 3.85%), *Fusobacterium periodonticum* (HOT-201; 3.01%), *Neisseria flavescens* (HOT-610; 2.94%), *Gemella sanguinis* (HOT-757, 2.60%), *Actinomyces graevenitzii* (HOT-866; 2.45%), *Haemophilus parainfluenzae* (HOT-718; 2.11%) and *Prevotella pallens* (HOT-714; 1.99%).

In summary, a total of 1429 OTUs were observed of which only 59 were represented by more than 0.02% of the reads, with eleven of these 59 mainly present in only one or two patients (Additional file 2 – grey colored cells) and 13 accounting for 65.03% of the reads.

### Comparison of the oropharyngeal microbial composition of the non-AZ-treated and AZ-treated patients

**Fig. 1:**
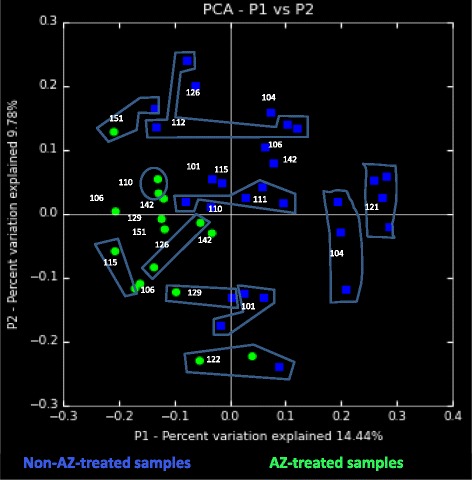
Beta diversity analysis of the oropharyngeal microbiomes of non-azithromycin-treated samples and azithromycin-treated samples. Legend: The Principal Coordinates Analysis plot using the phylogenetic-based unweighted UniFrac algorithm for the comparison of the oropharyngeal microbiomes of the non- azithromycin (AZ)-treated samples with the oropharyngeal microbiomes of the AZ-treated samples. Blue dots: non-AZ-treated samples (*n* = 28): all samples of the 5 placebo-receiving subjects (*n* = 20) + the V2 samples of the 8 AZ-treated subjects. Green dots: AZ-treated samples (*n* = 16): V3 and V6 samples of the 8 AZ-treated patients. Each group of dots of the same patient is labeled with the patient number

Beta diversity analysis, using the phylogenetic-based unweighted UniFrac algorithm Principal Coordinates Analysis, was applied to examine qualitative differences between the oropharyngeal microbiomes, based on presence or absence of OTUs in the non-AZ-treated samples (all 20 samples from the 5 placebo-receiving patients + V2 samples of the 8 AZ-treated patients, *n* = 28) versus the AZ-treated samples (visits V3 and V6 of the 8 AZ-treated patients, *n* = 16) (Fig. 1).

The oropharyngeal microbiomes of the AZ-treated samples clustered together (Fig. 1 and Additional file 3). Not only did the oropharyngeal microbiome of each treated patient shift to the left for Principal Coordinate 1 (capturing 14.44% of the variation) after the AZ treatment, the microbiomes of the AZ-treated samples also shifted closer to one another, which indicates that shared changes occurred for the treated samples. However, random Forest classification estimated error indicated that the treated and untreated samples were not significantly different from each other, because the estimated error rates were greater than 5% (Table 2).

**Table 2:** Supervised (machine) Learning Estimated Error Rates (randomForest simulation) for abundant OTU denoised pipelines

Clinical characteristic	Minimal Row Contribution Cut Off Sum for Each OTU			
	5	25	100	500
	Estimated Error Rate (%)			
Treatment (*i.e.* AZ-treated and non-treated)	15.91	13.64*	15.91	18.18
Visit (*i.e.* V2, V3, V6 and V7)	88.64	79.55	86.36	79.55
Patient group (*i.e.* Placebo, AZ responders, AZ non-responders)	22.73	25.0	20.45	20.45

Legend: Estimated error rate of the randomForest simulation by virtue of potentially contributable clinical metadata (Treatment, visit and Patient group) following an abundant OTU pipeline for denoising of dataset, *i.e.* following removal of chimeric and low quality sequences. Top row header: Minimal row contribution cut off sum for each OTU to determine the best performing data set (*i.e.* contains the most discriminative features with least amount of noise). When describing estimated error rate per minimal row, treatment was retained as the only clinical metadata category in the model simulation that had the smallest level of estimated error (closest to the < 10% mark for significance)

*: the cut off value of 25 reads was chosen for further analysis, because its error rate is closest to 10%

Fig. 1 also shows that several of the samples from the 5 placebo-receiving subjects (101, 104, 111, 112 and 121), whereby all of the samples represent untreated oropharyngeal microbiomes, cluster closely together per patient, indicating that the changes that are observed for the treated patients are not likely the result of random temporal changes in the oropharyngeal microbiome that could have occurred during the study period.

**Fig. 2:**
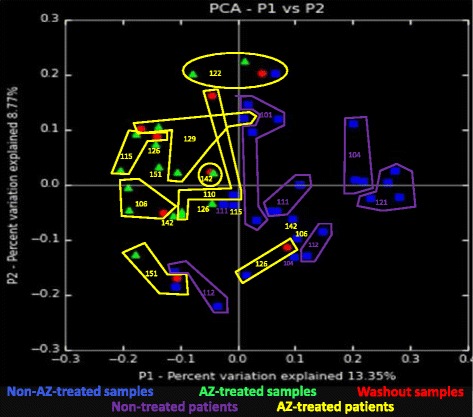
Beta diversity analysis of the oropharyngeal microbiomes of non-azithromycin-treated samples, azithromycin-treated samples and washout samples. Legend: The Principal Coordinates Analysis plot using the phylogenetic-based unweighted UniFrac algorithm for the comparison of the oropharyngeal microbiomes of the azithromycin (AZ)-treated samples (green triangles) with those of the non-AZ-treated samples (blue squares) and those of the washout samples of the 8 AZ-treated patients (red dots). Green triangles: AZ-treated samples: V3 and V6 of the 8 AZ-treated subjects (*n* = 16). Blue squares: non-AZ-treated samples: all 20 samples of the 5 placebo-receiving subjects and V2 samples from the 8 AZ-treated subjects (*n* = 28). Red dots: washout samples of the AZ-treated patients: V7 of the 8 AZ-treated patients (*n* = 8). Each group of dots of the same patient is labeled with the patient number. Group color corresponds to non-treated patients (purple), AZ-treated patients (yellow)

In addition, Fig. 2 shows that, for the 8 treated patients, 4 out of the 8 V7 samples (sampled one month after treatment) are more proximal to the untreated samples (V2) of the same patient than the during treatment samples V3 and V6, although the time lapse between V7 and V2 samples is larger than between V7 and V3-V6 samples. This similarity between pre- (V2) and post-treatment (V7) samples is suggestive of recovery of the original microbiome.

**Fig. 3:**
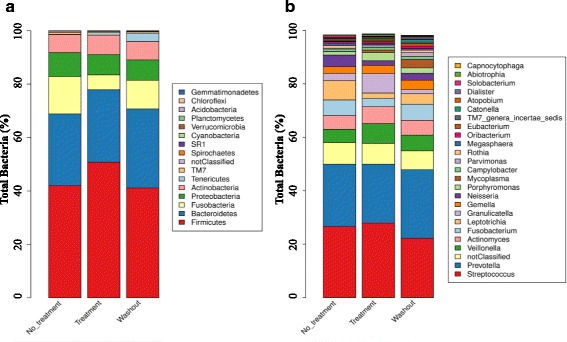
Taxonomic abundance comparison between azithromycin (AZ)-treated, non-AZ-treated and washout samples. Legend: Prevalence of the most abundant phyla **a** and genera (**b**) in the oropharyngeal cavity for the AZ-treated (*n* = 16), non-AZ-treated (*n* = 28) and washout (visit 7, after treatment, *n* = 8) samples. Taxonomic classification was made by means of the RDP classifier

When looking at the taxonomic composition of the microbiomes at the phylum level (Fig. 3), Firmicutes, Bacteroidetes, Fusobacteria, Proteobacteria and Actinobacteria appear to be the most abundant phyla, with little difference between the treated, untreated and washout samples. Fusobacteria decrease and Firmicutes increase after the start of and during AZ treatment, but their numbers virtually equal the pre-treatment status (V2) after a one month washout period (V7). It can be noticed that the phylum Tenericutes (more specifically the genus *Mycoplasma*) increases during treatment and even more so after the washout period, but this is a bias, mainly due to the observations in one patient (patient 122).

At the genus level, *Streptococcus* and *Prevotella* were the most abundant genera in both the treated and untreated samples (Fig. 3b). The genus *Fusobacterium* is more abundant in the pre-and post-treatment samples (Fig. 3b), which confirms the observations at the phylum level (Fig. 3a).

Crude analysis of the data, indicated that, of the 59 abundantly present OTUs, *Streptococcus salivarius* (the fifth most abundant species at 4.13%) increased in all 8 AZ-treated patients, with a median 4.9-fold increase, when comparing on-treatment (V3) with pretreatment (V2) samples. To the contrary, OTU numbers 942 and 1071, that both correspond to HOT entry 222, *i.e. Leptotrichia wadei* (the seventh most abundant species at 3.86%*)*, decreased in 7 out of the 8 AZ-treated patients, with a median 5.0-fold decrease.

In conclusion, the most prevalent species, except for *S. salivarius* and *L. wadei*, representing 81.76% of the oropharyngeal microbiome, were overall little affected by azithromycin treatment.

**Fig. 4:**
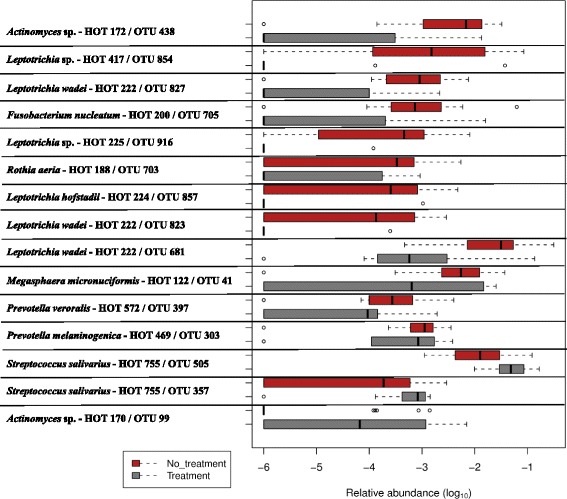
Relative abundance of bacterial taxa associated with the azithromycin (AZ)-treated vs. non-AZ-treated samples. Legend: The relative abundance refers to how common or rare a species is relative to other species in the oropharyngeal microbiome of the entire sample population (*n* = 44). Supervised (machine) learning with definition by randomForest and confirmation by Boruta feature selection (at cut off-value 25) enabled visualization of bacterial taxa associated with the AZ-treated (visits V3 and V6, *n* = 16) vs. non-AZ-treated samples (visit V2 from all samples and placebo samples, *n* = 28). The list is sorted first by Mann–Whitney U score followed by the largest disparity in medians between the No treatment group and the Treatment group. Taxa represent the lowest taxonomic depth that is labeled by RDP Classifier (at ≥ 80% bootstrap cut off). Boxes represent the first quartile, median, and third quartile of the distribution of OTUs for each treatment group. Empty circles represent outliers that are 1.5x greater than the respective interquartile ranges. The species level identification of the Boruta feature selection results were obtained through The Human Oral Microbiome Database (HOMD) Blast Search Tool

Boruta feature selection (Fig. 4) was used to look for features that differed significantly between the treated and untreated samples (at a cut off value of a minimum of 25 reads per OTU). A HOMD search was used to obtain species-level identification of the resulting OTUs (Additional file 4). Boruta feature selection confirms the conclusions that could be drawn from observing the raw data, namely that *S. salivarius* (HOT-755) increases due to AZ treatment (Fig. 4), whereas the species *L. wadei* (HOT-222) significantly decreases with AZ treatment. Also *Actinomyces* sp. (HOT-172), *Leptotrichia* spp. (HOT-417 and HOT-225), *Leptotrichia hofstadii* (and HOT-224) and *Fusobacterium nucleatum* (HOT-200) significantly decrease with AZ treatment (Fig. 4 and Additional file 4).

## Discussion

The oral microbiome has been shown to be the primary source of the healthy lung microbiome [18, 19] and indeed culture-independent studies have confirmed that the microbiome of the lungs more closely resembles that of the oropharynx than it does for instance that of other possible source communities, such as inhaled air and the nasopharynx [17–21]. This makes the oropharynx a valuable proxy for the lung microbiome, which requires less invasive techniques for sampling.

In this study, we assessed the microbiome composition of the oropharynx of 13 asthmatic patients and found the most abundant phyla to be the Firmicutes, Bacteroidetes, Fusobacteria, Proteobacteria and Actinobacteria (in descending order) and the most abundant genera to be *Prevotella* (*P. melaninogenica*: 15.17% of all reads, *P. pallens*: 1.99% and *P. nigrescens*: 1.42%) and *Streptococcus mitis* group (*S. mitis*/*pneumoniae*: 10.68%, *S. parasanguinis*: 6.84%, *S. infantis*: 0.95%, *S. australis*: 0.57%). *P. melaninogenica* and *S. mitis*/*pneumoniae* were the most abundant species, with both species together accounting for 25.8% of the reads. The asthma patients were colonized by the same phyla and genera that are commonly found in the oropharynx of healthy individuals, which has previously been characterized using culture-independent methods.

Lemon et al. [36] and Hilty et al. [37] determined the composition of the oropharyngeal microbiome of healthy individuals, using a gene clone library and found the *phyla Firmicutes, Proteobacteria and Bacteroidetes* to be the most prevalent. Lemon et al. [36] confirmed these findings with a 16S rRNA gene microarray. Also pyrosequencing of the 16S rRNA gene indicated that the healthy oropharynx is mainly colonized by the phyla Firmicutes, Actinobacteria, Bacteroidetes, Fusobacteria, candidate division TM7 and the genera *Actinomyces*, *Fusobacterium*, *Leptotrichia*, *Neisseria*, *Prevotella*, *Streptococcus* and *Veillonella* [38–41]. In addition, Segata et al. [40] showed that the microbiomes of saliva, tongue, tonsils, and throat (back wall of oropharynx) formed a group distinct from that of the microbiomes of the buccal mucosa/keratinized gingiva/hard palate group, the sub- and supra-gingival plaque group and the stool.

Hilty et al. [37] found the oropharyngeal microbial composition of healthy controls to be comparable to that of their asthmatic study population and Park et al. [42], using 16S rRNA gene pyrosequencing, observed no significant differences between asthma and COPD patients. Park et al. [42] reported that *Pseudomonas* spp. and *Lactobacillus* spp. were dominantly present in their asthmatic and COPD study population, but this was not the case in our study nor in that of Hilty et al. [37]. These different results could be due to a difference in the study populations, *i.e.* South Koreans [42] vs. Western populations (Hilty et al. [37], this study). The possibility of important geographical variation between oral microbiomes is illustrated by a study performed by Zaura et al. [43] where the salivary microbiomes were determined in a Swedish and a British population. Interestingly, the authors found that the predominant taxa in the Swedish saliva samples were *Prevotella* spp. and a *Porphyromonas* species, as opposed to *Streptococcus* spp. and *Rothia* spp. in the British saliva samples.

A recent oropharyngeal microbiome sequencing study by Castro-Nallar et al. [41] demonstrated that *Lactobacillus* spp., more specifically *L. gasseri*, were relatively more abundant in the oropharyngeal microbiome of schizophrenia patients, but overall they reported the presence of the same key members as established in other studies.

Interestingly, *Chlamydophila pneumoniae* and *Mycoplasma pneumoniae* were not recovered in this study or the studies mentioned above, although both species have been detected from patients with acute asthma exacerbations [6–8].

The effect of macrolides on the (oro) pharyngeal microbiome has been studied previously.

Malhotra-Kumar et al. [22] found that short-term treatment with macrolides, *i.e.* clarithromycin and azithromycin (AZ), induces a significant increase in macrolide-resistant pharyngeal streptococci in healthy volunteers. The COPD Clinical Research Network study [10] performed a randomized trial where the subjects were randomly assigned to receive AZ, at a dose of 250 mg daily (570 participants), or placebo (572 participants). They established an increased incidence of macrolide resistant streptococci in the nasopharyngeal microbiome as well. Recently, Zaura et al. [43] studied the effect of the widely used antibiotics clindamycin, ciprofloxacin, amoxycillin and minocycline on the salivary and fecal microbiome and found that the salivary microbiome is far more resilient toward the exposure to antibiotics than the fecal microbial community. Furthermore, the authors noted that minocycline, amoxycillin and clindamycin enhanced the prevalence of resistance genes, such as the erythromycin resistance methylase (*Erm*) and tetracycline efflux pump genes, in both the salivary and fecal microbiome [43]. These genes both cause resistance to the macrolides AZ and erythromycin [44].

In the present study, we assessed the effect of long term treatment (6 months) with AZ and the effects on microbiome composition one month after treatment. The phylum of the Firmicutes was positively affected by the AZ treatment and more specifically the species *Streptococcus salivarius* increased in AZ-treated samples. This confirms the observation in the study of Brusselle et al. [12], from which the samples for this study were collected, that long-term treatment with AZ was associated with an increased proportion of macrolide-resistant oropharyngeal streptococci (not further specified to the species level). In contrast, the phylum Fusobacteria, more specifically *Fusobacterium nucleatum* (HOT-200), and the genus *Leptotrichia*, more specifically *Leptotrichia* spp. (HOT-417 and HOT-225), *L. wadei* (HOT-222) and *L. hofstadii* (HOT-224), and *Actinomyces* sp. (HOT-172) were significantly decreased within the AZ-treated samples.

In the washout samples one month after the end of the treatment (V7 samples), it could be observed that the *Firmicutes* and *Fusobacteria* were evolving back to their pre-treatment status. Interestingly, the oropharyngeal microbiome of 4 of the 8 treated patients regained their initial composition within one month after the treatment. The present study has some weaknesses, although the number of samples (*n* = 52) was large, the number of patients (*n* = 13) was low, which could have influenced the random Forest classification estimated error rates (Table 2). However, there were no obvious differences in patient characteristics between the subjects included in this substudy as compared with those who were not included. There were no extraction controls and/or negative controls of the sampling procedure used in this study, which poses a possibility of contaminants remaining undetected and influencing the results obtained. However, our results of the non-treated samples concurred with those of earlier studies of the oropharyngeal microbiome [38–41].

## Conclusion

This study confirmed the overall composition of the oropharyngeal microbiome in patients with severe asthma and confirmed that this overall composition does not differ substantially from that of the healthy population. Only 13 species made up 65% of the deep sequencing reads, of which two (*Prevotella melaninogenica* and *Streptococcus mitis*/*pneumoniae*) accounted for 25,85% of the reads. We found that long term treatment (6 months) with azithromycin increased the species *Streptococcus salivarius* approximately 5-fold and decreased the species *Leptotrichia wadei* approximately 5-fold. Four of the 8 patients regained their initial composition within one month after cessation of the treatment. Long term azithromycin treatment may have long term effects on the composition of the oropharygeal microbiome in half of the patients, but this finding should be confirmed by further studies.

## Additional files

Additional file 1: Baseline characteristics for the AZISAST-trial (XLS 26 kb)

Additional file 2: Raw data, including read counts and sequences, of the 59 OTUs represented by 0.2% or more of the total number of reads. Species level identification was obtained through the HOMD database Blast search tool. (XLS 100 kb)

Additional file 3: Beta diversity analysis of the oropharyngeal microbiomes of the azithromycin-treated patients: visit 2 versus visit 3. Legend: The Principal Coordinates Analysis plot using the phylogenetic-based unweighted UniFrac algorithm for the comparison of the oropharyngeal microbiomes of the non- azithromycin (AZ)-treated samples with the oropharyngeal microbiomes of the AZ-treated samples. Blue dots: non-AZ-treated samples (*n* = 8): the Visit 2 samples (before treatment) of the 8 AZ-treated subjects. Green dots: AZ-treated samples (*n* = 8): Visit 3 samples (one month after the start of treatment) of the 8 AZ-treated patients. Each dot is labeled with the patient number and visit code. (PPTX 66 kb)

Additional file 4: Raw data, including read counts and sequences, of the 15 OTUs in Fig. 4 (bacterial taxa associated with the AZ-treated vs. non-AZ-treated samples) that resulted after Supervised machine learning. Species level identification was obtained through the HOMD database Blast search tool. (XLS 36 kb)

## Abbreviations

16S rRNA: 16S ribosomal Ribonucleic acid
ACQ: Asthma control questionnaire
AQLQ: Asthma quality of life questionnaire
AZ: Azithromycin
AZISAST: AZIthromycin in Severe ASThma (clinical trial)
BDP: Beclomethasone dipropionate
COPD: Chronic obstructive pulmonary disease
DNA: Deoxyribonucleic acid
emPCR: Emulsion PCR
*Erm*: Erythromycin resistance methylase
FeNO: Fractional exhaled nitric oxide
GINA: Global Initiative for Asthma
HOMD: Human Oral Microbiota Database
HOT: Human oral taxon
ICS: Inhaled corticosteroids
IQR: Interquartile range
LABA: Long-acting beta2-agonists
LRT: Lower respiratory tract
MID: Multiplex IDentifier
NCBI: National Center for Biotechnology Information
OTU: Operational Taxonomic Units
PcoA: Principal Coordinates Analysis
PCR: Polymerase chain reaction
QIIME: Quantitative Insights Into Microbial Ecology
RCT: randomized controlled clinical trial
RDP: Ribosomal Database Project
SFF: Standard flowgram format
V (2,3,6,7): Visit (2,3,6,7)

## References

[1] Cookson W (2004). The immunogenetics of asthma and eczema: a new focus on the epithelium. Nat Rev Immunol.

[2] Duan K, Dammel C, Stein J, Rabin H, Surette MG (2003). Modulation of *Pseudomonas aeruginosa* gene expression by host microflora through interspecies communication. Mol Microbiol.

[3] Goss CH, Burns JL (2007). Exacerbations in cystic fibrosis. 1: Epidemiology and pathogenesis. Thorax.

[4] Sibley CD, Parkins MD, Rabin HR, Duan K, Norgaard JC, Surette MG (2008). A polymicrobial perspective of pulmonary infections exposes an enigmatic pathogen in cystic fibrosis patients. Proc Natl Acad Sci U S A.

[5] Cardenas PA, Cooper PJ, Cox MJ, Chico M, Arias C, Moffatt MF (2012). Upper airways microbiota in antibiotic-naïve wheezing and healthy infants from the Tropics of Rural Ecuador. PLoS One.

[6] Biscione GL, Corne J, Chauhan AJ, Johnston SL. Increased frequency of detection of Chlamydophila pneumoniae in asthma. Eur Respir J. 2004;24:5. 10.1183/09031936.04.00049004 15516667

[7] Maffey AF, Barrero PR, Venialgo C, Fernandez F, Fuse VA, Saia M (2010). Viruses and atypical bacteria associated with asthma exacerbations in hospitalized children. Pediatr Pulmonol.

[8] Marri PR, Stern DA, Wright AL, Billheimer D, Martinez FD (2013). Asthma-associated differences in microbial composition of induced sputum. J Allergy Clin Immunol.

[9] Seemungal TA, Wilkinson TM, Hurst JR, Perera WR, Sapsford RJ, Wedzicha JA (2008). Long-term erythromycin therapy is associated with decreased chronic obstructive pulmonary disease exacerbations. Am J Respir Crit Care Med.

[10] Albert RK, Connett J, Bailey WC, Casaburi R, Cooper JA, Criner GJ (2011). Azithromycin for prevention of exacerbations of COPD. N Engl J Med.

[11] Brusselle GG, Joos GF, Bracke KR (2011). New insights into the immunology of chronic obstructive pulmonary disease. Lancet.

[12] Brusselle GG, Vanderstichele C, Jordens P, Deman R, Slabbynck H, Ringoet V (2013). Azithromycin for prevention of exacerbations in severe asthma (AZISAST): a multicentre randomised double-blind placebo-controlled trial. Thorax.

[13] Coeman M, van Durme Y, Bauters F, Deschepper E, Demedts I, Smeets P (2011). Neomacrolides in the treatment of patients with severe asthma and/or bronchiectasis: a retrospective observational study. Ther Adv Respir Dis.

[14] Friedlander AL, Albert RK (2010). Chronic macrolide therapy in inflammatory airways diseases. Chest.

[15] Crosbie PAJ, Woodhead MA (2009). Long-term macrolide therapy in chronic inflammatory airway diseases. Eur Respir J.

[16] Chung KF, Wenzel SE, Brozek JL, Bush A, Castro M, Sterk PJ, et al. International ERS/ATS guidelines on definition, evaluation and treatment of severe asthma. Eur Respir J. 2014;43:343–73. 10.1183/09031936.00202013 24337046

[17] Dickson RP, Huffnagle GB (2015). The lung microbiome: New principles for respiratory bacteriology in health and disease. PLoS Pathog.

[18] Venkataraman A, Bassis CM, Beck JM, Young VB, Curtis JL, Huffnagle GB (2015). Application of a neutral community model to assess structuring of the human lung microbiome. MBio.

[19] Bassis CM, Erb-Downward JR, Dickson RP, Freeman CM, Schmidt TM, Young VB (2015). Analysis of the upper respiratory tract microbiotas as the source of the lung and gastric microbiotas in healthy individuals. MBio.

[20] Morris A, Beck JM, Schloss PD, Campbell TB, Crothers K, Curtis JL (2013). Comparison of the respiratory microbiome in healthy nonsmokers and smokers. Am J Respir Crit Care Med.

[21] Segal LN, Alekseyenko AV, Clemente JC, Kulkarni R, Wu B, Chen H (2013). Enrichment of lung microbiome with supraglottic taxa is associated with increased pulmonary inflammation. Microbiome.

[22] Malhotra-Kumar S, Lammens C, Coenen S, Van Herck K, Goossens H (2007). Effect of azithromycin and clarithromycin therapy on pharyngeal carriage of macrolide-resistant streptococci in healthy volunteers: a randomised, double-blind, placebo-controlled study. Lancet.

[23] Guss AM, Roeselers G, Newton IL, Young CR, Klepac-Ceraj V, Lory S (2011). Phylogenetic and metabolic diversity of bacteria associated with cystic fibrosis. ISME J.

[24] Riehle K, Coarfa C, Jackson A, Ma J, Tandon A, Paithankar S (2012). The Genboree Microbiota Toolset and the analysis of 16S rRNA microbial sequences. BMC Bioinformatics.

[25] Genboree. http://www.genboree.org/site/. Accessed throughout 2015.

[26] Wang Q, Garrity GM, Tiedje JM, Cole JR (2007). Naïve Bayesian classifier for rapid assignment of rRNA sequences into the new bacterial taxonomy. Appl Environ Microbiol.

[27] Caporaso G, Kuczynski J, Stombaugh J, Bittinger K, Bushman F, Costello E (2010). QIIME allows analysis of high-throughput community sequencing data. Nat Methods.

[28] Lozupone C, Knight R (2005). UniFrac: a new phylogenetic method for comparing microbial communities. Appl Environ Microbiol.

[29] Liaw A, Wiener M (2002). Classification and Regression by randomForest. R news.

[30] Kursa MB, Rudnicki WR (2010). Feature selection with the Boruta package. JStatSoft.

[31] Aagaard K, Riehle K, Ma J, Segata N, Mistretta TA, Coarfa C (2012). A metagenomic approach to characterization of the vaginal microbiome signature in pregnancy. PLoS One.

[32] HOMD - Human Oral Microbiome Database. http://www.homd.org/. Accessed 5 Dec 2016.

[33] Chen T, Yu WH, Izard J, Baranova OV, Lakshmanan A, Dewhirst FE. The Human Oral Microbiome Database: a web accessible resource for investigating oral microbe taxonomic and genomic information. Database, Vol. 2010, Article ID baq013, doi: 10.1093/database/baq013. 10.1093/database/baq013 PMC2911848 20624719

[34] Dewhirst FE, Chen T, Izard J, Paster BJ, Tanner AC, Yu WH (2010). The human oral microbiota. J Bacteriol.

[35] National Center for Biotechnology Information – BioProject. https://www.ncbi.nlm.nih.gov/bioproject/PRJNA356972. Accessed 21 Jan 2016.

[36] Lemon KP, Klepac-Ceraj V, Schiffer HK, Brodie EL, Lynch SV, Kolter R (2010). Comparative analyses of the bacterial microbiota of the human nostril and oropharynx. MBio.

[37] Hilty M, Burke C, Pedro H, Cardenas P, Bush A, Bossley C (2010). Disordered microbial communities in asthmatic airways. PLoS One.

[38] Charlson ES, Bittinger K, Haas AR, Fitzgerald AS, Frank I, Yadav A (2011). Topographical continuity of bacterial populations in the healthy human respiratory tract. Am J Respir Crit Care Med.

[39] Huttenhower C, Gevers D, Knight R, Abubucker S, Badger JH, Chinwalla AT, et al. Human Microbiome Project Consortium. Structure, function and diversity of the healthy human microbiome. Nature. 2012;486:207–14. 10.1038/nature11234 PMC3564958 22699609

[40] Segata N, Haake SK, Mannon P, Lemon KP, Waldron L, Gevers D (2012). Composition of the adult digestive tract bacterial microbiota based on seven mouth surfaces, tonsils, throat and stool samples. Genome Biol.

[41] Castro-Nallar E, Bendall ML, Pérez-Losada M, Sabuncyan S, Severance EG, Dickerson FB (2015). Composition, taxonomy and functional diversity of the oropharynx microbiome in individuals with schizophrenia and controls. PeerJ.

[42] Park H, Shin JW, Park SG, Kim W (2014). Microbial communities in the upper respiratory tract of patients with asthma and chronic obstructive pulmonary disease. PLoS One.

[43] Zaura E, Brandt BW, Teixeira de Mattos MJ, Buijs MJ, Caspers MPM, Rashid MU, et al.Same exposure but two radically different responses to antibiotics: resilience of the salivary microbiome versus long-term microbial shifts in feces. mBio. 2015;6:e01693–15. 10.1128/mBio.01693-15 PMC4659469 26556275

[44] Phuc Nguyen MC, Woerther PL, Bouvet M, Andremont A, Leclercq R, Canu A (2009). *Escherichia coli* as reservoir for macrolide resistance genes. Emerg Infect Dis.

