# Supplementary figures and images for: Influence of chronic azithromycin treatment on the composition of the oropharyngeal microbial community in patients with severe asthma
Source: BMC Microbiol. 2017 May 10;17:109. doi: 10.1186/s12866-017-1022-6 (PMC5424369; doi:10.1186/s12866-017-1022-6)

## Slide 1
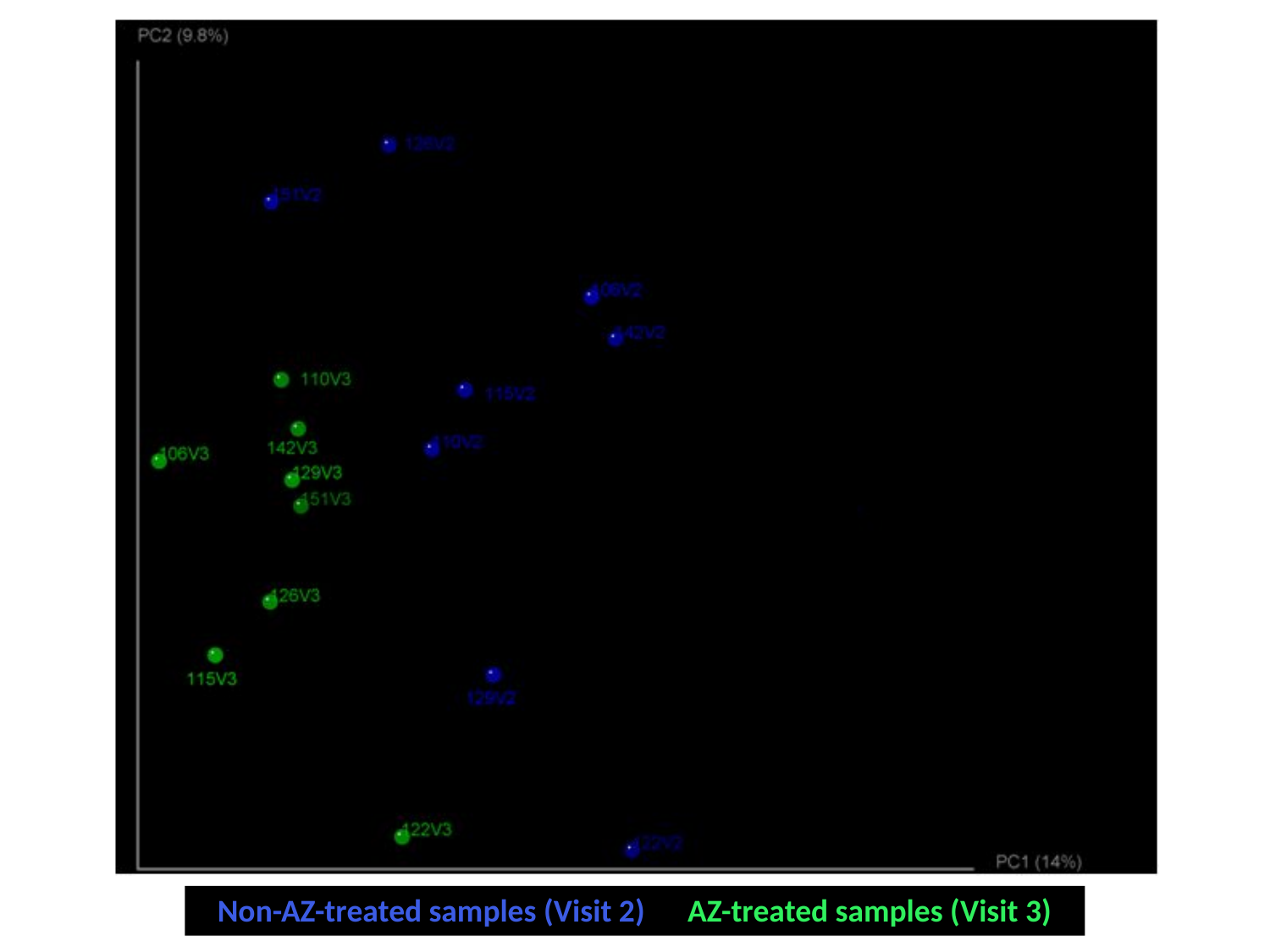

Non-AZ-treated samples (Visit 2)
AZ-treated samples (Visit 3)

Supplement: Supplementary file 3 — Beta diversity analysis of the oropharyngeal microbiomes of the azithromycin-treated patients: visit 2 versus visit 3. Legend: The Principal Coordinates Analysis plot using the phylogenetic-based unweighted UniFrac algorithm for the comparison of the oropharyngeal microbiomes of the non- azithromycin (AZ)-treated samples with the oropharyngeal microbiomes of the AZ-treated samples. Blue dots: non-AZ-treated samples (n = 8): the Visit 2 samples (before treatment) of the 8 AZ-treated subjects. Green dots: AZ-treated samples (n = 8): Visit 3 samples (one month after the start of treatment) of the 8 AZ-treated patients. Each dot is labeled with the patient number and visit code. (PPTX 66 kb) [file 12866_2017_1022_MOESM3_ESM.pptx]
